# Electroencephalography in Normotensive and Hypertensive Pregnancies and Subsequent Quality of Life

**DOI:** 10.1371/journal.pone.0155299

**Published:** 2016-05-11

**Authors:** Ingrid A. Brussé, Johannes J. Duvekot, Ivette Meester, Gerard Jansen, Dimitris Rizopoulos, Eric A. P. Steegers, Gerhard H. Visser

**Affiliations:** 1 Department of Obstetrics and Gynaecology, Division of Obstetrics and Prenatal Medicine, Erasmus MC, University Medical Centre, Rotterdam, the Netherlands; 2 Department of Haematology, Erasmus MC, University Medical Centre, Rotterdam, the Netherlands; 3 Department of Biostatistics, Erasmus MC, University Medical Centre, Rotterdam, the Netherlands; 4 Department of Clinical Neurophysiology, Erasmus MC, University Medical Centre, Rotterdam, the Netherlands; 5 SEIN- Epilepsy Institute in the Netherlands, Heemstede, the Netherlands; University of Barcelona, SPAIN

## Abstract

**Objectives:**

To compare electroencephalography (EEG) findings during pregnancy and postpartum in women with normotensive pregnancies and pregnancies complicated by hypertensive disorders. Also the health related quality of life postpartum was related to these EEG findings.

**Materials and Methods:**

An observational case-control study in a university hospital in the Netherlands. Twenty-nine normotensive and 58 hypertensive pregnant women were included. EEG’s were recorded on several occasions during pregnancy and 6–8 weeks postpartum. Postpartum, the women filled out health related quality of life questionnaires. Main outcome measures were qualitative and quantitative assessments on EEG, multidimensional fatigue inventory, Short Form (36) Health Survey and EuroQoL visual analogue scale.

**Results:**

In women with severe preeclampsia significantly lower alpha peak frequency, more delta and theta activity bilaterally and a higher EEG Sum Score were seen. Postpartum, these women showed impaired mental health, mental fatigue and social functioning, which could not be related to the EEG findings.

**Conclusions:**

Severe preeclamptic patients show more EEG abnormalities and have impaired mental wellbeing postpartum, but these findings are not correlated.

## Introduction

Preeclampsia (PE) is a multi-organ disease of unknown cause unique to human pregnancy. PE affects 1–7% of all pregnancies. Neurologic complications due to hypertensive disease in pregnancy are among the leading causes of maternal morbidity and mortality. [1]

Currently we do not have a tool to monitor disturbances in the brain function in order to prevent complications. Electroencephalography (EEG) is known to be a sensitive method to detect brain dysfunction. EEG changes can be found before clinical symptoms are present and before ischemic conditions may result in irreversible brain dysfunction. [2] We have published a systematic review on EEG in hypertensive pregnancy and believe there is need for new data, using the current terminology and definitions. [3] The objective of this review was to evaluate the available medical literature concerning the EEG during hypertensive disorders of pregnancy. Abnormal EEG findings were observed in the majority of the women with PE/eclampsia, consisting of slow waves most frequently localized in the occipital lobe, as well as spike discharges. The EEG abnormalities in PE/eclampsia were reversible in the majority of the cases. We concluded that the described abnormalities might be interpreted as a warning sign of deterioration of brain function in PE/eclampsia. However, we advised some caution regarding this conclusion, because most of the retrieved articles were published in the 1950s and 1960s, and were not consistent with current clinical guidelines or medical terminology.

Somatic symptoms of PE, such as hypertension and proteinuria, generally disappear rapidly after delivery. However, formerly preeclamptic women more often complain of mental health problems as compared to women after uncomplicated pregnancies. [4] These complaints may persist and can interfere with quality of life. Therefore, these complaints must not be underestimated.

We hypothesized that severe PE influences the brain through hypoxia and edema. This could therefore have an effect on brain function, which does not disappear immediately after delivery. This altered brain function could influence physical and mental health. In this study we describe EEG during the course of normotensive pregnancies as compared to hypertensive pregnancies and we relate EEG findings to health related quality of life (HRQoL) 6 to 8 weeks postpartum.

## Materials and Methods

In this prospective longitudinal case-control study normotensive healthy pregnant women, serving as a control group, and patients with chronic hypertension (CH), pregnancy induced hypertension (PIH), mild preeclampsia (mild PE) and severe preeclampsia (severe PE) underwent EEG testing during their pregnancies and postpartum. Postpartum all women filled out HRQoL questionnaires.

The Medical Ethics Committee Erasmus MC of Rotterdam approved the study protocol (MEC-2005-142). After obtaining written informed consent, participants were included in this study from October 2005 till October 2008.

Normotensive women, women with CH and women with PIH were consecutively recruited in the outpatient clinic. Patients with mild PE and severe PE were recruited after admission at the department of obstetrics of the Erasmus MC. Normotensive women and those with CH were examined at the following gestational ages: 12–14 weeks, 26–28 weeks, 32–34 weeks and 36–40 weeks. Women who developed PIH were included when diagnosed and examined at the same time points. Preeclamptic patients were consecutively included at admission and, if possible, measured weekly until delivery. Severely preeclamptic patients underwent an EEG after initial stabilization with antihypertensive medication and, if necessary, magnesium sulphate. Finally, all participants were re-examined 6–8 weeks postpartum. All participants spoke Dutch fluently. Exclusion criteria were: use of medication other than antihypertensive medication and/or magnesium sulphate, gestational diabetes, diabetes mellitus, pre-existing neurological disorders and psychiatric illnesses.

According to the criteria of the International Society for the Study of Hypertension in Pregnancy (ISSHP), PIH was defined as de novo hypertension after 20 weeks of gestation in absence of proteinuria and PE as hypertension in the presence of de novo proteinuria. [5] Severe PE was diagnosed if in addition one or more of the following criteria were present: a systolic blood pressure of 160 mmHg or higher and/or a diastolic blood pressure of 110 mmHg or higher; proteinuria of 5 gram or more in a 24-hour urine specimen or dipstick urinalysis of 3+ or greater in two random urine samples collected at least 4 hours apart; oliguria of less than 500 mL in 24 hours; cerebral or visual disturbances; pulmonary oedema or cyanosis; epigastric or right upper-quadrant pain; impaired liver function; thrombocytopenia; foetal growth restriction. [6] All patients who did not meet these criteria for severe PE were diagnosed as having mild PE.

Ethnicity and the level of education were obtained from the questionnaires. Level of education was assessed by the highest completed education. It was divided into three categories: low (primary school, lower vocational training and intermediate general school), mid (intermediate and higher vocational training) and high (university degree). [7]

**Fig 1:**
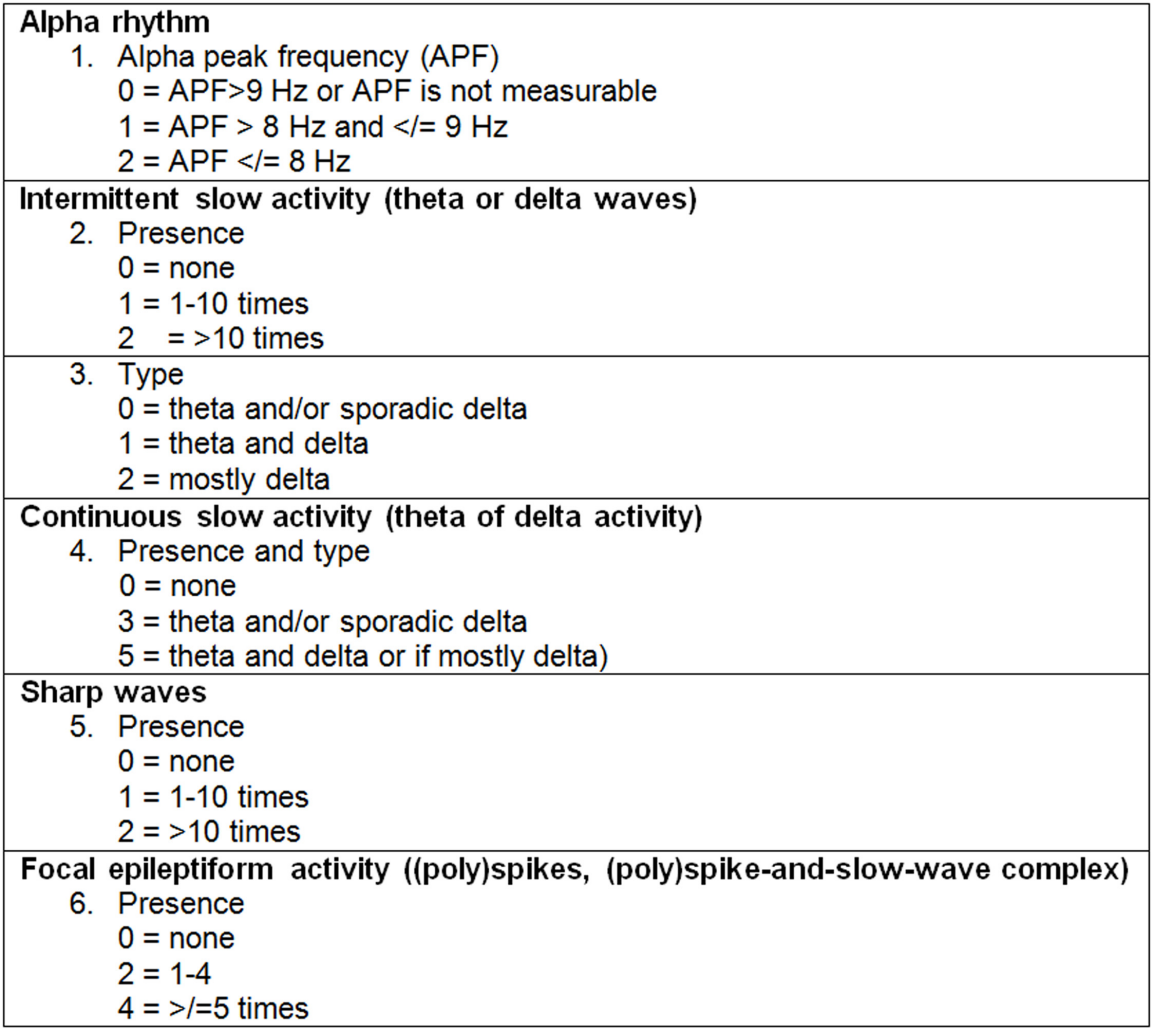
EEG Sum Score ^a^. ^a^ EEG Sum Score is the sum of items 1 to 6, ranging 0–17 points.

The EEG recordings were made in accordance with the 10–20 International System of Electrode Placement. Electrode impedances were less than 5 kΩ. [8] The registrations were recorded during wake. EEG registrations were blinded reviewed by one of the authors (GHV) using referential, source, and bipolar montages. In addition, the dominant occipital EEG activity (alpha rhythm) was quantitatively assessed using fast Fourier Transformation (FFT) and documented for both sides of the brain as alpha peak frequency (APF) (samples with epoch length 10 s; ≥ 10 epochs averaged). The EEG alpha rhythm is electrical activity with an occipital maximum that can be seen in relaxed persons with eyes closed. In a healthy adult the normal frequency is above 8.5 Hz. Reference values of the APF are not gender specific and show a mean of 10.02 Hz (SD 0.9). [9] The visual assessment scored presence, type, amount and localization of intermittent slow activity, continuous slow activity, sharp waves and focal epileptiform activity. Delta waves are slow frequency waves present in deep sleep. Theta waves are slow frequency waves present just before sleep. We subdivide interictal findings in sharp waves and focal epileptiform activity. Sharp waves are associated with epileptic activity, but can also been found in people who never develop epileptic seizures. Focal epileptic activity always contains complexes with spikes and slow waves. Definitions of terms in interictal findings are published in Appendix S4 in Epilepsia. [10] These activities were combined in the EEG Sum Score (Fig 1). This score was adapted from the Grand Total EEG score, which was published by Jonkman ranging from score 0 for a completely normal EEG to 17 for the most severely abnormal EEG. [11, 12].

HRQoL is a multidimensional concept with physical, psychological and social domains. The combination of the domain-specific Multidimensional Fatigue Inventory (MFI) for fatigue and two generic measures of own health, Short Form (36) Health Survey (SF-36) and the EuroQoL visual analogue scale (EQ-VAS) classification, show good psychometric performance, are internationally standardized and widely used in the postpartum period. [13–15]

For statistical analysis SPSS was used, version 20.0 (SPSS Inc. Chicago, Illinois, US). *P*-values < 0.05 were considered significant. The differences between the study groups on baseline characteristics and HRQoL were analysed using ANOVA, Pearson Chi-Square tests and Fisher’s exact tests when applicable. ANCOVA was used to study the association of HRQoL with covariates. The correlation between the EEG items and HRQoL items was computed using the Spearman’s correlation coefficient. To perform this analysis for the APF during pregnancy we used the area under the longitudinal trajectory of each patient as a summary of her APF profile (i.e., average APF during pregnancy). Postpartum we used the postpartum value of the APF. To account for the correlation in the repeated measurements of each patient during pregnancy, a repeated measures analysis has been performed to analyse each parameter using linear mixed effects models. Accordingly, for the Sum Score, a Poisson mixed effects regression was utilized. To allow for possible nonlinearities in the parameters’ evolution in time we used regression splines of time in the specification of the fixed and random effects of the model, when supported from the data. The repeated measures analysis has been performed in the R statistical software (version 2.15.1) using package nlme (version 3.1–104). The models’ assumptions were checked using residuals plots.

## Results

A total of 103 pregnant women were initially included. Fifteen patients withdraw their participation after the first examination and one patient miscarried, remaining 87 study subjects: 29 normotensive women, nine CH, six PIH, 14 mild PE and 29 severe PE patients. Two of the previously normotensive pregnant patients developed PE (one mild PE and one severe PE) and one other patient developed PIH. They were categorized and analysed in the group with their contracted hypertensive disease. A total of 85 women received the HRQoL questionnaires, 71 questionnaires were returned. In 66 of those 71 cases an EEG was made postpartum.

Table 1 shows the patient characteristics. As expected, there are significant differences between the five groups in blood pressure, gestational age, mode of delivery and parity.

**Table 1:** Patient characteristics.

Characteristics	Normotensive (n = 29)	Chronic hypertension (n = 9)	Pregnancy Induced Hypertension (n = 6)	Mild Preeclampsia (n = 14)	Severe Preeclampsia (n = 29)	P value
Age (years) ^a^^,^^b^	32.0 (5.3)	32.5 (3.2)	32.0 (5.1)	32.2 (4.5)	30.3 (6.5)	.687
Systolic blood pressure (mmHg) ^a^^,^^b^	113 (12.1)*^†^^‡^	122 (16.5)*^†^	136 (16.9)	136 (10.2)	145 (16.8)	.000^#^
Diastolic blood pressure (mmHg) ^a^^,^^b^	65 (7.1)*^†^^‡^^§^	78 (8.0)*^†^^‡^	87 (9.8)	87 (4.8)	92 (10.2)	.000^#^
Gestational age al delivery (days) ^a^^,^^b^	277(11.5)*^†^	273 (12.3)*	266 (15.0)*	257 (19.4)*	224 (29.3)	.000^#^
Level of education ^c^^,^^d^						.004^#^
*Low*	10.7	12.5	50	0	0	
*Mid*	60.7	75	16.7	92.9	85.2	
*High*	28.6	12.5	33.3	7.1	14.8	
Ethnicity ^c^^,^^d^						.763
*Caucasian*	82.8	77.8	66.7	78.6	69.0	
*Non-Caucasian*	17.2	22.2	33.3	21.4	31	
Mode of delivery ^c^^,^^d^						.000^#^
*Vaginal*	82.8	100	83.3	50.0	20.7	
*Elective cesarean*	13.8	0	16.7	21.4	75.9	
*Emergency cesarean*	3.4	0	0	28.6	3.4	
Parity ^c^^,^^d^						.027^#^
*Nulliparous*	51.7	11.1	50.0	50	62.1	
*Para-1*	37.9	88.9	16.7	42.9	27.6	
*Para-2*	3.4	0	0	7.1	20.3	
*Para-3*	6.9	0	33.3	0	0	
NICU admission (days) ^f^	0 (0–2)	0 (0–5)	0 (0–0)	2.5 (0–35)	13 (0–92)	.000^#^^,^^e^
Perinatal death ^g^	0	0	0	0	1	

^a^ Mean (SD)

^b^ One way ANOVA was used

^c^ Number (%)

^d^ Chi-Square test (Fisher’s exact) was used

^e^ Kruskal-Wallis test was used

^f^ Total

^g^ Median (range)

* p≤ 0.05 vs. sPE,

^†^p≤ 0.05 vs. mPE,

^‡^p≤ 0.05 vs. PIH,

^§^p≤ 0.05 vs. CH

^#^ p≤ 0.05

Abbreviations: NICU, Neonatal Intensive Care Unit; sPE, severe preeclampsia; mPE, mild preeclampsia; PIH, pregnancy induced hypertension; CH, Chronic Hypertension

**Fig 2:**
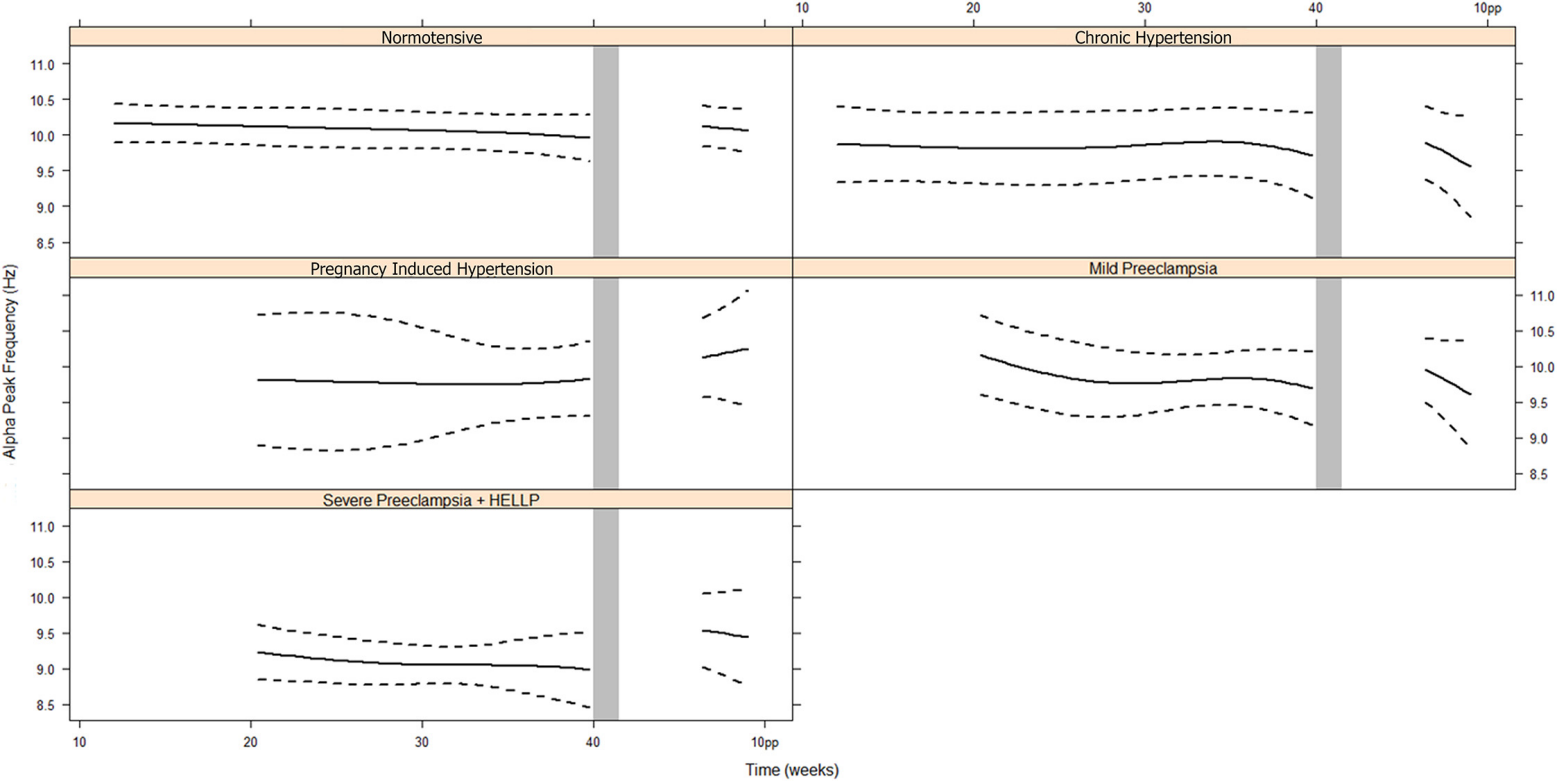
Alpha Peak Frequency during pregnancy and post partum ^a^. ^a^ The repeated measures analysis has been performed using linear mixed effects models. Data are presented in mean and 95% Confidence Interval. Abbreviations: Hz, Hertz; pp, post partum, HELLP, Hemolysis Elevated Liver enzymes Low Platelets.

Fig 2 presents APF during pregnancy and postpartum for each group.

In 5 normotensive women, during one or more of the measurements, no alpha rhythm was detected and therefore APF could not be determined. In the groups with CH, PIH, mild PE and severe PE there were respectively 1, 0, 2 and 2 women where the APF could not be determined during one or more of the measurements. These measurements were excluded for APF analysis. The APF was measured occipital on the left and right side at every time point of the measurements. There was no significant left-right difference in APF in the normotensive group (Intraclass Correlation Coefficient of > 0.930 at all 5 time points). Therefore we used the mean of the right and left APF in further analysis. There was a strong intra-individual correlation between all subsequent time points (all pair wise r> 0.864, p< 0.001).

We found no evidence of a time effect (Likelihood Ratio Test (LRT) = 13.5, degrees of freedom (d.f.) = 15, *p* = 0.560), meaning that the APF remained constant during pregnancy. However, there was an overall statistical difference between the five groups (LRT = 38.3, d.f. = 20, *p* = 0.008), but there was no indication that this difference attributed to differences in the longitudinal evolutions (LRT = 8.2, d.f. = 12, *p* = 0.770). The severe PE group differed significantly from the other groups (versus normal: *p* = 0.000, versus CH: *p* = 0.058, versus PIH: *p* = 0.058, versus mild PE: *p* = 0.007). The APF was on average 0.6 Hz higher postpartum than it was during pregnancy (*p* = 0.001) in all groups, but there were no significant differences between this increase and the absolute values postpartum (LRT = 2.6, d.f. = 4, *p* = 0.629).

There is no evidence of association between the mean APF and systolic blood pressure (*p* = 0.692), or between APF and diastolic blood pressure (*p* = 0.229). Repeated measures ANOVA did not show a significant age-effect (*p* = 0.191) or an effect of parity (*p* = 0.500) on the APF values.

The amount, type and localization of EEG abnormalities are shown in Table 2. Per person more than one abnormality can be found. More intermittent slow and continuous slow activity was seen in the mild PE and severe PE groups, especially bilateral delta and theta activity fronto-temporal. In a few cases of severe PE sharp waves were seen and in one case epileptiform activity was seen postpartum. In the severe PE group abnormalities were more frequently. Because we did not find unilateral continuous slow activity and unilateral epileptiform activity, these activities were not included in Table 2.

**Table 2:** Visually assessed EEG.

Abnormal activity	Normotensive		Chronic Hypertension		Pregnancy Induced Hypertension		Mild Preeclampsia		Severe Preeclampsia	
	Pregnancy (n = 29)	Postpartum(n = 27)	Pregnancy (n = 9)	Postpartum (n = 8)	Pregnancy (n = 6)	Postpartum (n = 6)	Pregnancy (n = 14)	Postpartum (n = 13)	Pregnancy (n = 29)	Postpartum (n = 25)
Intermittent slow activity ^a^	8	7	2	3	1	0	5	3	16	15
Type										
*Theta*	5	5	1	2	0	0	1	2	8	7
*Theta + sporadic delta*	3	1	1	0	0	0	3	1	4	5
*Theta + delta*	0	1	0	1	1	0	1	0	4	2
*Delta*	0	0	0	0	0	0	0	0	0	1
Hemisphere										
*Unilateral*	1	1	0	1	0	0	0	0	0	0
*Bilateral*	7	6	2	2	1	0	5	3	16	15
Localization										
*Diffuse*	0	1	0	0	0	0	1	0	3	1
*Focal*	F = 1,FT/T = 7	FT/T = 6	FT/T = 2	FT/T = 2,C/P = 1	C/P = 1	0	FT/T = 4	FT/T = 3	F = 1,FT/T = 8,O = 1	F = 4,FT/T = 8,C/P = 1
*Multifocal*	0	0	0	0	0	0	0	0	2	1
Continuous slow activity ^a^	1	1	2	0	0	0	3	0	8	3
Type										
*Theta*	1	1	2	0	0	0	2	0	7	2
*Theta +sporadic delta*	0	0	0	0	0	0	1	0	0	1
Hemisphere										
*Bilateral*	1	1	2	0	0	0	3	0	7	3
Localization										
*Diffuse*	0	0	0	0	0	0	0	0	5	2
*Focal*	FT/T = 1	0	FT/T = 2	0	0	0	0	0	FT/T = 1	FT/T = 1
*Multifocal*	0	1	0	0	0	0	3	0	1	0
Focal sharp waves ^a^	1	1	1	2	0	0	0	1	1	3
Hemisphere										
*Unilateral*	1	0	0	0	0	0	0	0	0	1
*Bilateral*	0	1	1	0	0	0	0	1	1	2
Localization										
*Focal*	FT/T = 1	FT/T = 1	FT/T = 1	FT/T = 2	0	0	0	FT/T = 1	O = 1	F = 1,FT/T = 2
Focal epileptiform activity ^a^	0	0	0	0	0	0	0	0	0	1
Hemisphere										
*Bilateral*	0	0	0	0	0	0	0	0	0	1
Localization										
*Focal*	0	0	0	0	0	0	0	0	0	FT/T = 1

^a^ Total

Abbreviations: F, frontal; FT/T, fronto-temporal; C/P, central or parietal; O, occipital

**Fig 3:**
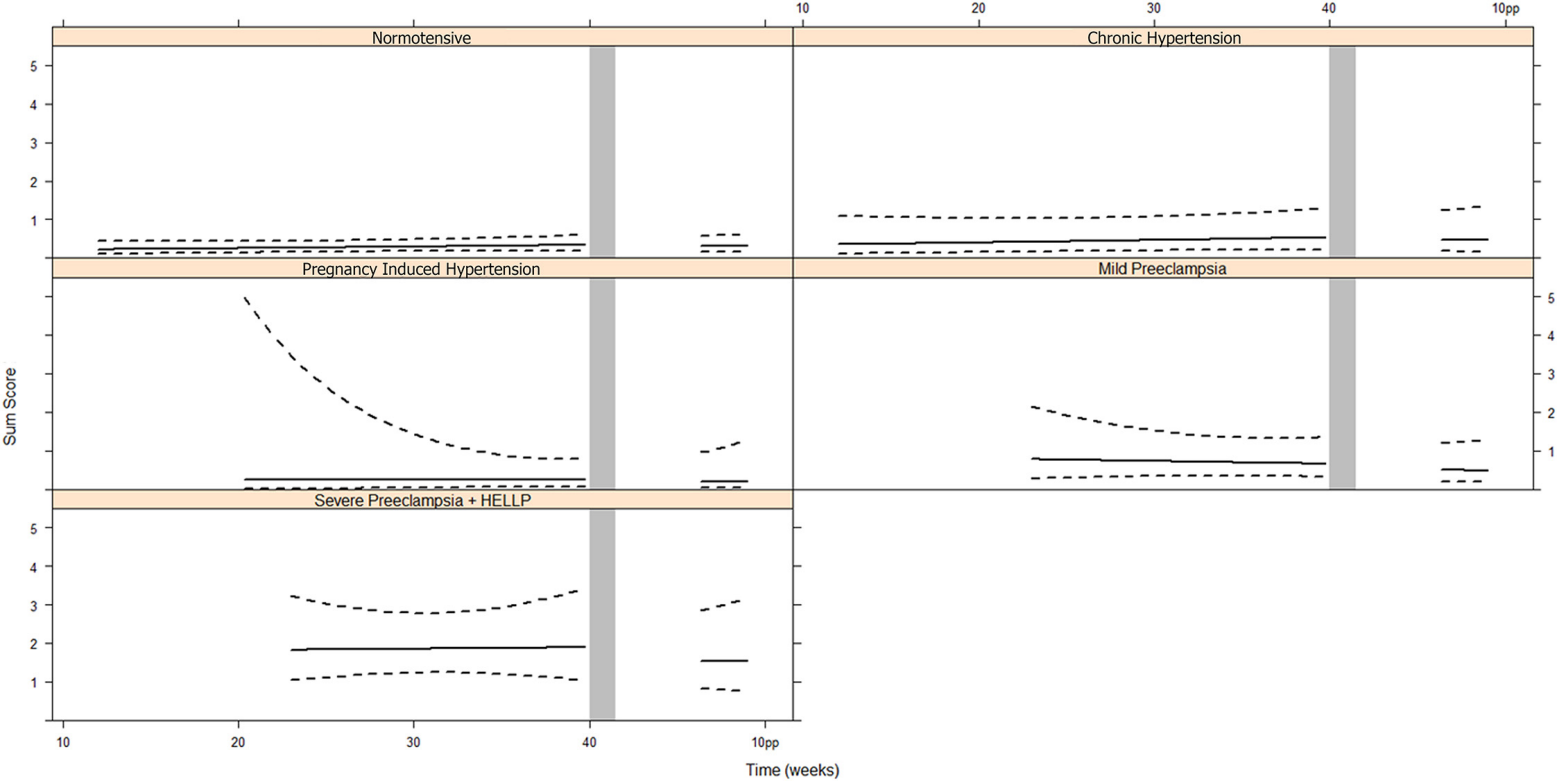
Sum Score during pregnancy and post partum ^a^. ^a^ The repeated measures analysis has been performed using linear mixed effects models. Data are presented in mean and 95% Confidence Interval. Abbreviations: pp, post partum, HELLP, Hemolysis Elevated Liver enzymes Low Platelets.

Fig 3 presents the EEG Sum Score during pregnancy and postpartum for each group. We found no evidence of a time effect (LRT = 3.8, d.f. = 5, *p* = 0.581), meaning that the Sum Score also remained constant during pregnancy. There was an overall statistically significant difference between the five groups (LRT = 40.3, d.f. = 12, *p*<0.0001), but there was no indication that this difference attributed to the differences in the longitudinal evolutions (LRT = 2.8, d.f. = 4, *p* = 0.594). The severe PE group differed significantly from the other groups (versus normal: *p* = 0.000, versus CH: *p* = 0.027, versus PIH: *p* = 0.005, versus mild PE: *p* = 0.043). The mean Sum Score was on average 0.213 lower postpartum than it was during pregnancy and was not statistically different between the groups (*p* = 0.360). We did not find statistical differences between the study groups in the postpartum measurement of the EEG Sum Score (LRT = 3.2, d.f. = 4, *p* = 0.525). Furthermore, we found no evidence of an association between the EEG Sum Score and systolic blood pressure (*p* = 0.624) or diastolic blood pressure (*p* = 0.445).

**Fig 4:**
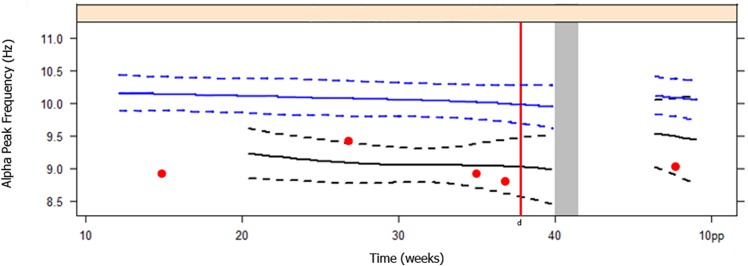
Course of Alpha Peak Frequency in a patient who developed Severe Preeclampsia ^a,b,c^. ^a^ Data of patient is presented in red dots. ^b^ Black line and dotted lines are data of Severe Preeclampsia + HELLP, as presented in Fig 2. ^c^ Blue line and dotted lines are data of Normotensive women, as presented in Fig 2. ^d^ Red line: patient developed Severe Preeclampsia. Abbreviations: Hz, Hertz; pp, post partum; HELLP, Hemolysis Elevated Liver Enzymes Low Platelets.

In our study one woman in the normotensive group developed severe PE. Her individual APF values were plotted in Fig 4. She developed severe PE in the 38^th^ week of gestation. This woman had a relatively low APF during her entire pregnancy and postpartum. The visual assessment of her EEG showed no anomalies, except at a gestational age of 35 weeks, as it showed focal bilateral intermittent theta and sporadic delta activity, which can be classified as mild and non-specific abnormalities. The other two normotensive women, who developed to PIH and mild PE, had APF values within the ranges of these groups.

Table 3 shows the results of the HRQoL questionnaires (EQ-VAS, MFI and SF-36). Items reflecting mental health and fatigue, and social functioning were statistically different between the study groups. The patients with severe PE showed the worst HRQoL scores.

**Table 3:** Results of EQ-VAS. MFI and SF-36.

	Normotensive (n = 25)	Chronic Hypertension (n = 8)	Pregnancy Induced Hypertension (n = 6)	Mild Preeclampsia (n = 9)	Severe Preeclampsia (n = 23)	Cronbach’s alpha	P value ^a^ (unadjusted)	P value ^b^ (adjusted)
VAS (score 0–100) ^c^	83.3 (10.7)*	85.3 (5.8)	84.3 (12.4)	81.7 (12.0)	76.5 (11.2)		0.165	0.294
MFI (score 4–20) ^c^								
MFI total	47.6 (16.8)*	40.4 (14.6)*	42.5 (16.8)*	41.4 (11.1)*	57.3 (17.4)		0.038^#^	0.149
General Fatigue	11.8 (3.9)	10.5 (3.5)	11.5 (3.3)	11.6 (3.6)	13.3 (4.5)	0.86	0.465	0.740
Physical Fatigue	8.9 (4.5)	6.9 (2.5)*	10.0 (4.9)	8.6 (2.7)	10.9 (4.1)	0.89	0.149	0.429
Mental Fatigue	9.4 (4.3)*	8.6 (5.5)	6.8 (4.7)*	6.2 (2.1)*	12.0 (5.1)	0.92	0.012^#^	0.003^#^
Reduced Motivation	7.9 (4)	6.7 (2.2)	6 (2.5)*	7.1 (2.7)	9.4 (3.7)	0.80	0.137	0.248
Reduced Activity	9.6 (4.1)	7.6 (4.4)*	8.2 (5.1)	8.0 (3.2)*	11.3 (3.6)	0.83	0.097	0.402
SF-36 (score 0–100) ^c^								
Physical Sum Score	51.0 (8.6)	52.0 (3.6)	46.3 (9.3)	50.7 (7.1)	47.9 (8.3)		0.455	0.638
Physical Functioning	87.1 (14.7)	93.0 (7.0)	80.0 (19.0)	95.0(5.0)	97.0 (10.5)	0.79	0.141	0.467
Role Physical	75.0 (36.9)	90.6 (26.5)	70.8 (33.2)	86.1 (33.3)	64.1 (36.0)	0.82	0.319	0.181
Bodily Pain	74.4 (30.9)	78.5 (29.7)	66.0 (28.5)	74.9 (29.8)	66.3 (30.8)	0.78	0.802	0.185
General Health	84.3 (13.4)*	82.0 (10.7)*	82.8 (13.9)*	79.3 (12.2)	70.4 (14.0)	0.51	0.010^#^	0.249
Mental Sum Score	50.4 (8.4)	56.5 (6.0)*	56.5 (5.3)*	55.6 (3.5)*	46.2 (10.7)		0.006^#^	0.020^#^
Vitality	60.2 (19.3)	73.6 (11.3)*	65.0 (21.7)	62.2 (16.0)	52.2 (19.2)	0.76	0.068	0.236
Social Functioning	78.5 (19.3)^†^^§^	95.3 (6.5)*	89.6 (12.3)*	93.1 (12.7)*	68.5 (21.6)	0.80	0.001^#^	0.022^#^
Role Emotional	83.3 (31.1)	98.5 (35.4)	94.4 (13.6)	96.3 (11.1)	72.5 (41.0)	0.84	0.326	0.153
Mental Health	78.7 (14.0)	90.5 (8.8)*	84.0 (16.0)	88.4 (8.6)*	72.7 (15.9)	0.75	0.008^#^	0.001^#^

^a^ One way ANOVA was used

^b^ ANCOVA was used to correct for maternal age, ethnicity, level of education, parity, gestational age and mode of delivery, and admission of the neonate at the NICU

^c^ Mean (SD)

* p≤ 0.05 vs. sPE,

^†^p≤ 0.05 vs. mPE,

^§^p≤ 0.05 vs. CH

# p< 0.05

Abbreviations: NICU, Neonatal Intensive Care Unit; sPE, Severe Preeclampsia; mPE, Mild Preeclampsia; PIH, Pregnancy Induced Hypertension; CH, Chronic Hypertension

The internal consistency of the SF-36 and the MFI multi-item scales was determined with Cronbach’s alpha-coefficient. The item General Health in the SF-36 had a Cronbach’s alpha-coefficient of 0.51, due to the fact that the answers to some of the questions were given inconsistently. The other items had a Cronbach’s alpha-coefficient of 0.70 or higher. Based on this, the internal consistency of the SF-36 and the MFI multi-item scales was considered sufficient for the purpose of group comparisons.

We investigated the association between the APF and EEG Sum Score values prior to delivery and postpartum with the HRQoL items measured postpartum. As a summary of the mean AFP during the course of pregnancy we used the area under the longitudinal trajectory of each patient. As a summary of the EEG Sum Score during the course of pregnancy we used the area under the longitudinal trajectory of each patient. The Spearman correlation coefficients between the summaries of the longitudinal trajectories and the postpartum HRQoL items, the APF values and EEG Sum Scores did not show any significant correlation.

All items in Table 3 were tested for confounders. Differences between the study groups remained significant for the items mental fatigue (*p* = 0.003), mental sum score (*p* = 0.020), social functioning (*p* = 0.022) and mental health (*p* = 0.001) correcting for maternal age, ethnicity, level of education, parity, gestational age and mode of delivery, and admission of the neonate to the neonatal intensive care unit (NICU).

## Discussion

This is the first longitudinal study on both quantitative and qualitative EEG findings in normotensive pregnant women and women with hypertensive disorders of pregnancy. In women with severe PE significantly lower APF, more bilateral delta and theta activity and a higher EEG Sum Scores were seen. These findings recovered postpartum. Postpartum, these women showed more impaired mental health, mental fatigue and decreased social functioning. These HRQoL outcomes, however, were not correlated with the above-mentioned EEG changes, as we hypothesized.

Our study shows that the APF remains constant during the course of pregnancy in all groups. In patients with severe PE the APF was significantly lower during the clinical phase of the disease. The APF was already altered during the non-clinical phase in the only normotensive patient that developed severe PE (Fig 4), but postpartum the APF remained low. The latter may be explained by a pre-existing phenomenon or by slow recovery from severe PE. Postpartum the differences in APF between the study groups disappeared, reflecting recovery of this aspect of brain function in severe PE patients. In only three patients with severe PE we recorded APF lower than 8.5 Hz during pregnancy. Postpartum, their APF measurements returned within the normal range. Low values in APF have also been found in patients with psychiatric disorders like depression, chronic fatigue syndrome and burnout. [16] A decrease in APF is related to decreased performance on memory tasks. [17] In 2008 we published a study that showed that memory was impaired in formerly severe preeclamptic women. [18]

We were not able to stratify for early (n = 5) and late onset severe PE (n = 24). In the subgroup of patients with early onset severe PE, APF was 0.8 Hz lower. However, this was not significant different, probably because of small sample size, but could be indicative for worse clinical disease. There were no significant differences in APF for the other hypertensive groups. The confidence intervals for the CH en PIH groups are wide (Fig 2), probably caused by the small sample size. We could not identify alpha rhythm during one or more of the sessions in 10 women (11.5%). In other studies the APF is poorly visualized in up to 25% of the general population due to inability to relax. [19]

The EEG abnormalities found in normotensive pregnancies were mild, showing intermittent slow mostly theta activity and occasionally sharp waves, predominantly in the frontal and temporal areas (Table 2). Focal epileptiform activity was not seen in the normotensive group. In women with abnormalities these were both seen during pregnancy and postpartum. In severe PE multifocal and bilateral intermittent and continuous slow activity was seen more frequently. Postpartum the decrease in continuous slow activity can be regarded as an improvement of the supposed hypertensive encephalopathy in these patients, although there was some increase in sharp waves and epileptiform activity. We can only speculate whether these abnormalities were already present before pregnancy, pregnancy induced, or will remain permanently. The EEG abnormalities found in this study might be reflected in cognitive failures like memory loss, as was earlier described. [18, 20] Persistent cerebral anomalies were described in a MRI study in both formerly eclamptic and preeclamptic women with neurological symptoms and may actually reflect permanent brain ischemia. [21] In our study EEG anomalies subsided or disappeared 6–8 weeks postpartum. EEG is in our opinion a more precise and sensitive instrument to detect functional brain damage than MRI.

In order to evaluate the visually assessed EEG in combination with the APF we summarized the EEG characteristics in the EEG Sum Score. This score did not significantly vary during the course of pregnancy and postpartum. Even the individual scores remained constant. This could either mean that the neuronal activity in the brain is not normalized completely 6 to 8 weeks postpartum, or is not influenced by pregnancy. Combining this finding with the results on APF, the first opinion is most likely.

We found in one third of the normotensive women (n = 9) that the EEG Sum Score was more than 0 during the first measurement, due to minor abnormalities in the visual assessment. Literature on EEG in normotensive pregnancies is scarce and relatively old. [3] Two relatively small studies report EEG abnormalities in 15% of normotensive pregnant women. [22, 23] Volume shifts, blood pressure and hormonal changes could be responsible. It’s tempting to speculate that these minor EEG abnormalities reflect the often reported cognitive disturbances like mental slowness and forgetfulness during uneventful pregnancies.

The number of studies on the long term effects of PE is increasing. [20, 24–26] As somatic symptoms merely disappear after delivery, many women continue to complain of cognitive disturbances. We showed that the severe PE group has the worst HRQoL scores, significantly in items reflecting mental fatigue, mental health and social functioning. These results are in line with other studies. Postpartum maternal morbidity after preeclamptic pregnancy may be directly related to the severity. [27, 28] The psychosocial impact, admittance to an intensive care unit and health of the newborn may also affect women’s mental health. [24, 29, 30]

Lower APF and visual EEG abnormalities resulting in higher EEG Sum Score are mostly seen in the severe PE group during pregnancy. These abnormalities did not correlate with worse HRQoL outcomes postpartum. Probably other, unknown, factors of the disease not evident in the EEG could have had more impact on HRQoL postpartum.

The strength of our study is the presence of longitudinal data on both quantitative and qualitative EEG findings during normotensive and hypertensive pregnancies, and postpartum. Another strength is that we quantified EEG data in APF and introduced the EEG Sum Score.

Due to the low incidence of preeclampsia a very large amount of normotensive women should be included to be able to perform EEGs systematically prior to the onset of PE. This can be seen as a limitation of our study. We chose to compare EEGs in a group of normotensive pregnant women to women with hypertensive disorders of pregnancy. Unfortunately, we included relatively small sample sizes in the groups of women with CH en PIH.

Our data can be used as reference values for EEGs performed during pregnancy and in the postpartum period. Preconception and late postpartum measurements are lacking which could be considered as a limitation, but logistically this was not possible.

This study adds knowledge of the reduced wellbeing that might be helpful in providing care for women after severe PE, which has consequences for their postpartum return to normal life and work.
